# Incidence and Management Trends in Advanced Head and Neck Non-Melanoma Skin Cancer in Ontario

**DOI:** 10.3390/curroncol33050291

**Published:** 2026-05-17

**Authors:** Ka-Kit David Yeung, Gregory Pond, Isaac Kong, Han Zhang, Michael Gupta, Zejia Chen, Justin Lee

**Affiliations:** 1 Division of Radiation Oncology, Department of Oncology, Faculty of Health Sciences, McMaster University, Hamilton, ON L8S 4L8, Canada; 2 Division of Otolaryngology, Department of Surgery, Faculty of Health Sciences, McMaster University, Hamilton, ON L8S 4L8, Canada; 3 Faculty of Medicine, Queen’s University, Kingston, ON K7L 3N6, Canada

**Keywords:** head and neck cancer, non-melanoma skin cancer, adjuvant radiation, radiation oncology, adjuvant treatment, population-based study, Canada, Ontario

## Abstract

Head and neck non-melanoma skin cancers (H&N NMSCs) account for most head and neck malignancies. While primary treatment comprises surgery, adjuvant radiation is recommended in advanced tumors. Radiation oncology practice patterns for resected locally advanced (rLA) and locoregional (rLR) H&N NMSCs have not been well characterized. Using data from the Institute for Clinical Evaluative Sciences (ICES) between 2003 and 2019, we conducted a longitudinal, population-based study characterizing disease incidence, survival outcomes, and radiation utilization patterns. Among 2962 rLA and 1055 rLR cases, rLA incidence rose more than tenfold compared to population growth; however rLR incidence remained stable. Radiation oncology consultations occurred in 29.6% of rLA and 50.7% of rLR patients. Increased age, multiple cancers at diagnosis, non-rural demographic, and higher SES were observed to be correlated to receipt of adjuvant radiation treatment. Only 19.4% of rLA and 37.95 of rLR disease received adjuvant RT, which is much lower than expected based on international guidelines. Five-year overall survival (OS) was 69% (95% confidence interval (CI): 67–71%) for rLA and 68% (95% CI: 65–71%) for rLR disease. These findings highlight the burden of advanced H&N NMSC, low rates of radiation utilization and the need for improving referral pathways and guideline adherence.

## Simple Summary

Non-melanoma head and neck skin cancers are the most common cancers in the head and neck region. For people with advanced or more widespread disease, the American Society of Radiation Oncology has published guidelines that recommends surgery to be followed by radiation to lower the risk of cancer recurrence. However, it is unclear how often patients are referred to a radiation oncologist or receive this treatment. Using Ontario health data from 2003 to 2019, this study included more than 4000 patients with these cancers to characterize disease rates and identify factors influencing radiation referral and treatment. The number of patients with advanced disease grew over tenfold faster than population growth, but only 35% of those with locally advanced or nodal disease had a consultation with a radiation specialist. These findings highlight a need to understand why referrals and utilization are low and may inform the implementation of care pathways and local guidelines.

## 1. Introduction

Non-melanoma skin cancers (NMSCs) are the most prevalent type of cancer in North America, making up 28% of new cancer cases in Canada with an estimated 36% increase in incidence since the 2000s [1,2,3]. Over 77% of these cases are basal cell carcinomas (BCCs), with the remaining 23% being squamous cell carcinomas (SCCs) [4,5,6]. Of these, 80% of cases occur in the head and neck area, classified as head and neck non-melanoma skin cancers (H&N NMSCs) [7,8,9,10,11,12,13].

Most patients have early-stage disease with a cure rate of over 99% in basal cell carcinomas and over 90% in squamous cell carcinomas [11,14,15,16]; however at least 4% of patients present with locally advanced (T3-4 N0 M0 disease) or locoregional node-positive disease (T1-4 N1-3 M0 disease), which is associated with a 5 year overall survival (OS) of less than 50% for squamous cell carcinomas [14,16,17,18,19,20,21]. Surgical excision is considered the primary curative treatment approach; however, recent studies suggest that adjuvant radiotherapy is associated with improved recurrence-free survival (RFS) and OS in selected high-risk populations [2,22,23,24,25,26,27,28].

Dermatology guidelines highlight the need for radiation oncology input in cutaneous squamous cell carcinoma patients, and recommended multidisciplinary consultation with consideration of adjuvant radiation therapy (RT) for patients with perineurial invasion (PNI), lymph node metastasis or high-risk features for metastatic spread [29]. The American Society for Radiation Oncology (ASTRO) guidelines similarly recommend postoperative radiation for locally advanced cutaneous SCCs with PNI, T3 or T4 staging, positive margins post-resection, recurrent disease, or lymph node metastases with or without extranodal extension. ASTRO also recommends adjuvant radiation in basal cell carcinoma with PNI or nodal involvement and conditional treatment for primary basal cell carcinomas with close or positive margins, recurrent disease or infiltration into adjacent bone or muscle [2].

While these recommendations exist, the frequency of radiation oncology involvement after radical surgical resection has not been studied, and it is unclear what proportion of H&N NMSCs with high-risk features receive adjuvant RT. This population-based study was conducted to present a longitudinal analysis to highlight radiation oncology utilization patterns and survival outcomes in resected locally advanced (rLA) and locoregional (rLR) H&N NMSCs. This study will present the methods used to identify the incidence of advanced, resected H&N NMSCs in Ontario over a 17-year period, followed by a description of patient characteristics, clinical outcomes and analysis of radiation oncology consultation and utilization in this patient population.

## 2. Materials and Methods

### 2.1. Data Source

This study included adult patients without prior radiation treatment or surgeries with a corresponding International Classification of Diseases version 10 (ICD-10) diagnosis code of H&N NMSC (Appendix A) in Ontario, Canada. Data was collected from 2003 to 2019, inclusive. The end date of 2020 was chosen to mitigate the impact of the global pandemic of COVID-19. Ontario is the largest province in Canada, representing approximately 14 million people. Several healthcare administrative databases housed and de-identified by the Institute for Clinical Evaluative Sciences (ICES) were linked and used to collect radiation oncology utilization patterns and patient outcome data. These databases report information on patients in Ontario covered by the Ontario Health Insurance Plan (OHIP), the provincial insurance plan that covers all Ontario citizens, permanent residents and full-time foreign workers in the province. Datasets included were (1) the Ontario Cancer Registry, (2) the Registered Persons Database, which reports information on patient characteristics and OS, (3) the National Ambulatory Care Reporting system, which reports patient data for day surgery and outpatient clinics, (4) the New Drug Funding Program database, which reports cancer treatment data, (5) the OHIP claims database, which includes all physician billings for cancer-related care; all physicians must bill OHIP for cancer-related care they provide in Ontario’s single-payer public healthcare system.

To identify patients with rLA and rLR disease, disease states were inferred from the American Joint Committee on Cancer (AJCC) 8th edition staging manual. Tumors classified as T3-4 N0 M0 were used as a surrogate for locally advanced (LA) disease, and tumors designated with any T stage N1-3 M0 were used as a surrogate for locoregional (LR) disease. Staging information available is based on Cancer Care Ontario’s ‘best stage’ grouping approach, using the pathologic Tumor, Node, Metastasis classification system (TNM) if available, and clinical TNM if not [30]. Corresponding rLA and rLR disease stages were identified using OHIP physician billing codes following pathologic diagnosis (Appendix A). Early-stage disease patients were also included in the analysis for comparison and were identified by including OHIP billing codes showing T1-2 N0 M0 staging compared to rLA or rLR disease. Utilizing surrogates for staging allows for the possibility of misclassification bias. This method of classifying stage was used because the ICES databases do not contain pathologic data. Other pathologic risk factors such as histologic subtype, PNI, margin status, or extranodal extension were also not available for data collection.

Inclusion criteria included adult patients with ICD-10 codes of C44.0, C44.1, C44.2, C44.3, and C44.4 (Appendix B), treated with surgical resection as defined by billing codes (Appendix A) in Ontario, Canada, between 2003 and 2019, inclusive. Exclusion criteria were stage 0 disease, non-H&N histologies, and patients who received radiation or surgery to the head and neck area more than 30 days prior to the diagnosis.

Demographic and tumor information influencing patients’ ability to receive RT was collected. Characteristics included age at diagnosis, sex, Charlson Comorbidity Index, Ontario Marginalization Index, income quintile, Rural Index Ontario, histology of index cancer, and history of previously diagnosed malignancy. OS and RFS of patients were collected.

Recurrence metrics were defined as any cause of death or repeat LA- or LR-associated surgery more than 30 days post surgery. Surgical billing codes (Appendix A) within this timeframe were used to identify the repeat resections.

Patients were deemed as assessed using radiation oncology for RT if there was an OHIP billing code for radiation oncology consultation within 60 days after the stage-defining surgery. Adjuvant radiation was defined as any radiation treatment OHIP billing code within 90 days from the stage-defining surgery.

### 2.2. Statistical Analysis

Descriptive statistics were used to summarize patient and treatment characteristics, as well as outcomes between staging groups.

Incidence of disease was investigated to identify disease burden in Ontario and was calculated as the number of new cases per year. Change in incidence per year was estimated using linear regression. Frequency of consultations and treatments over time was calculated as the proportion of patients having a consultation or receiving treatment, divided by the number of patients diagnosed for each year. The Cochran–Armitage test for trend was used to investigate whether treatment patterns changed over time.

Logistic regression analysis was used to investigate factors prognostic for receipt of adjuvant radiation, and Cox’s regression analysis was used to investigate the association of adjuvant radiation on OS. To avoid the effect of survivorship bias, a landmark analysis of 90 days was used to correspond with the definition of adjuvant radiation. A multivariable model was constructed using all available factors provided by the databases, and the impact of adjuvant radiation was evaluated accounting for variables in the model. All tests and confidence intervals were two-sided, and statistical significance was defined at the 0.05 level.

## 3. Results

### 3.1. Descriptive Statistics

A total of 11,308 cases were associated with the ICD-10 code of H&N NMSC from 2003 to 2019. Of these, 150 patients were excluded due to prior radiation and 1405 patients were excluded due to H&N NMSC-associated surgery prior to 2003. Resected early-stage disease was the most common with 4111 cases identified, followed by rLA with 2962 cases and rLR with 1055 cases. Some patients may have had multiple synchronous or metachronous H&N NMSCs. Patients across resected early-stage disease, rLA, and rLR had a male prevalence of 62.7%, 65.6%, and 67.9%, respectively. Based on the ICD-10 codes, other and unspecified parts of the face (not lip, eyelid, ear, scalp, or neck) were the most common anatomic location for early-stage, rLA and rLR groups. Patient characteristics are described in Table 1.

**Figure 1:**
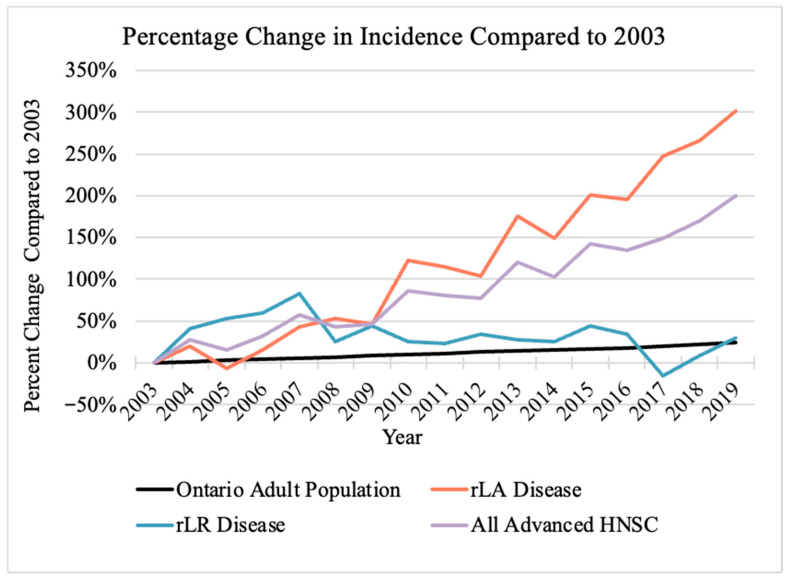
Percentage change in incidence in head and neck non-melanoma skin cancer cases compared to Ontario population growth. Resected locally advanced disease (rLA) is depicted by red line; resected locoregional (rLR) disease is depicted by blue line; all advanced head and neck skin cancers (HNSCs) are depicted by purple line; and Ontario population is depicted by black line.

The incidence of rLA H&N NMSC increased over time with a mean increase of 14.9% cases per year (95% confidence interval (CI): 13.1%, 16.7%; *p* < 0.001). rLR disease had no significant change in incidence with an estimated change of −0.81% cases per year (95% CI: −1.79%, 0.18%; *p* = 0.13). The percent increase in rLA, rLR, and all advanced H&N NMSCs relative to baseline population growth is shown in Figure 1.

rLA disease was associated with a 5-year RFS of 52% (95% CI: 49% to 54%). For patients who initially had rLA disease, when looking at any recurrence over time, 16.9% had local recurrences, 3.9% had regional recurrences, 4.1% had both local and regional recurrences, and 25.4% of patients had deaths without prior diagnosis of recurrence. rLR disease had a total 5-year RFS of 54% (95% CI: 51% to 57%). For patients with initial rLR disease, when looking at any recurrence over time, 9.2% had local recurrences, 7.1% had regional recurrences, 5.8% had both local and regional recurrences and 31.2% had deaths without prior diagnosis of recurrence. Mortality data was all-cause, and not cancer-specific. The disease state of either rLA or rLR disease was not associated with a statistically significant HR for any recurrence over time with a HR of 0.95 (95% CI: 0.85, 1.06; *p* = 0.37). The 5-year OS was 69% (95% CI: 67, 71) for rLA disease and 68% (95% CI: 65, 71) for rLR disease (Table 2).

### 3.2. Predictive Factors for Overall Survival with Landmark Date of 90 Days

Univariate and multivariate analysis of OS from a landmark date of 90 days was performed. Multivariate analysis showed that adjuvant radiation in rLA disease had a hazard ratio (HR) for OS of 1.67 (95% CI: 1.45, 1.92; *p* < 0.001). The HR for adjuvant radiation in rLR disease on OS was 2.57 (95% CI: 2.05, 3.21; *p* < 0.001).

For rLA disease, female sex was associated with better OS with a HR of 0.77 (95% CI: 0.67, 0.89; *p* < 0.001). Increased age was associated with worse OS outcomes in rLA disease with a HR of 1.46 (95% CI: 1.41, 1.51; *p* < 0.001). For rLA disease, primary disease of the lip (ICD-10 code C44.0) was associated with the highest HR compared to disease in other areas of the head and neck.

Similarly, for rLR disease, female sex had a HR of 0.73 (95% CI: 0.58, 0.91; *p* = 0.006) and increased age had a HR of 1.25 (95% CI: 1.20, 1.30; *p* < 0.001). Primary disease of the eyelid (ICD-10 code C44.1) was associated with the highest relative HR. However, this was not statistically significant. The HR for rLR disease was 1.14 (95% CI: 1.01, 1.30; *p* = 0.039) compared to rLA disease on multivariate analysis (Table 3). This indicates that rLR had poorer survival when compared to rLA disease.

### 3.3. Predictive Factors for Radiation Oncology Consultation (Prior to or Within 60 Days of Surgery)

**Figure 2:**
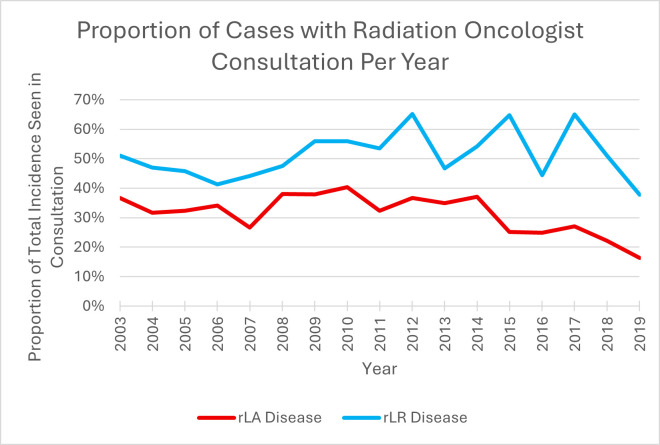
Proportion of new head and neck non-melanoma skin cancer cases that received radiation oncology consultation (prior to surgery or within 60 days following surgery) over time. Resected locally advanced disease (rLA) is depicted by red line and resected locoregional (rLR) disease is depicted by blue line.

Overall, 29.6% of patients with rLA disease and 50.7% of patients with rLR disease received a radiation oncology consultation prior to or within 60 days of surgery (Table 1). Figure 2 summarizes the trends in the proportion of radiation oncology consultation. The proportion of patients with rLA and rLR disease who received a radiation oncology consultation did not have a statistically significant trend with a mean yearly change of 1.2% (95% CI: −4.0%, 1.6%, *p* = 0.41) and 1.3% (95% CI: −3.9%, −1.2%, *p* = 0.32), respectively.

For prognostic factors for radiation oncology consultation, the multivariate analysis results show that for rLA disease, increased age and multiple cancers at diagnosis were associated with an odds ratio (OR) of 1.07 (95% CI: 1.04, 1.11; *p* < 0.001) and 4.61 (95% CI: 2.58, 8.26; *p* < 0.001), respectively. For rLR disease, increased age and multiple cancers at diagnosis had ORs of 1.09 (95% CI: 1.04, 1.13; *p* < 0.001) and 3.45 (95% CI:1.57, 7.57; *p* = 0.002), respectively. Primary disease of the lip (ICD-10 code C44.0) was also prognostic of radiation oncology consultation compared to other codes in rLA disease; however this was not found for rLR disease (Table 4). rLR disease in general was associated with an increased OR of receiving a radiation oncology consultation compared to rLA disease according to multivariate analysis with a value of 1.94 (95% CI:1.64, 2.29; *p* < 0.001).

### 3.4. Prognostic Factors for Receiving Adjuvant Radiation

**Figure 3:**
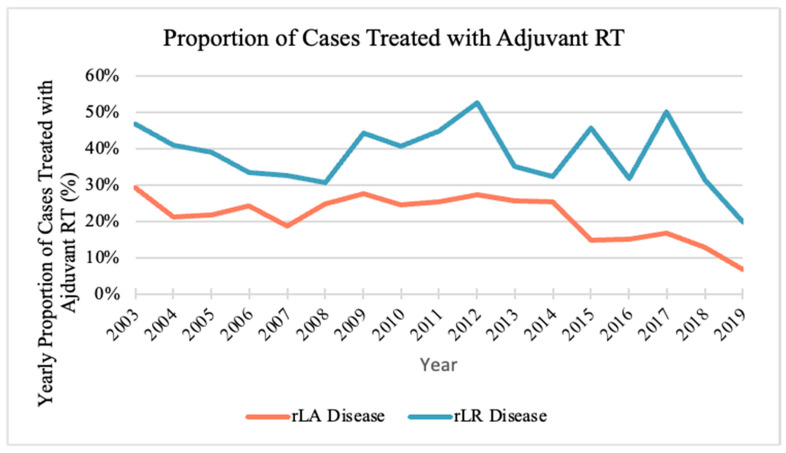
Proportion of new head and neck non-melanoma skin cancer cases treated with adjuvant radiation therapy (RT). Resected locally advanced disease (rLA) is depicted by red line and resected locoregional (rLR) disease is depicted by blue line.

The proportion of patients with rLA and rLR disease who received adjuvant radiation decreased with time, with mean changes of −1.6% (95% CI: −4.3%, 1.1%, *p* = 0.26) and −1.6% (95% CI: −5.0 to 1.9, *p* = 0.39), respectively, but this was not statistically significant (Figure 3). Over the 17-year period, the percentage of patients with rLA disease who received radiation therapy was 19.4% and with patients with rLR it was 37.9%.

Multivariate analysis was performed to find factors prognostic of adjuvant RT. In both rLA or rLR disease, increased age, multiple cancers at diagnosis, and non-rural demographic (defined as <100 km to nearest cancer center) was associated with an increased OR of receiving adjuvant radiation of 1.08 (95% CI: 1.05, 1.12; *p* < 0.001), 2.20 (95% CI: 1.36, 3.56; *p* = 0.001), and 1.52 (95% CI: 1.09; 2.12, *p* = 0.013), respectively. rLR disease was also associated with an increased OR for adjuvant radiation compared to rLA disease with an OR of 2.85 (95% CI: 2.27, 3.57; *p* < 0.001) (Table 5).

## 4. Discussion

Patterns of practice in advanced H&N NMSC including SCCs and BCCs have not been well characterized. While surgical excision is the first-line standard of care, current guidelines suggest that adjuvant radiation can improve OS and RFS outcomes in locally advanced tumors and tumors with locoregional spread. Using multiple administrative databases this study assessed patterns of practice and outcomes of more than 4000 patients who completed surgery for locally advanced and/or node-positive skin cancer over a period of seventeen years in the province of Ontario.

### 4.1. Incidence and Population Demographics

When combining the incidence of rLA and rLR disease, the incidence of H&N NMSC increased over time. This is attributed to the disproportionate rise in rLA disease incidence. rLA disease incidence grew over the study period, increasing yearly at a rate of 14.9%, far exceeding the 1.37% yearly population growth of Ontario in the same time frame [31]. However, rLR disease incidence was not found to significantly change year-to-year. While BCCs have low rates of nodal or distant metastases, SCCs with more advanced primary tumors are expected to have higher rates of nodal mestastases. While we were unable to assess histologic subtype in this study, future histologic analysis could shed light on the discrepancy between the incidence rates of rLA and rLR. The age of presentation and increasing incidence of rLA without growth in rLR disease may reflect the efforts of skin cancer screening and public education, leading to earlier detection and potentially prior to nodal metastatic disease progression. The increase in non-melanoma skin cancers in Ontario is consistent with previously reported trends [12], and is also consistent with the aging Ontario population as H&N NMSC is more prevalent in the later decades of life due to greater cumulative sun exposure [32].

### 4.2. Survival Outcomes

The 5-year OS of 69% for rLA disease and 68% for rLR disease is similar to that reported by other studies conducted in the United States [4,5,6]. The HR for OS in rLR disease was 1.14 compared to rLA disease, which aligns with previous literature suggesting LR disease is associated with poorer outcomes [14,16,17,18,19,20,21,22]. It is critical to note that differences in overall survival observed in patients receiving adjuvant RT or adjuvant systemic therapy are not indicative of a causal relationship and more likely reflect biases in the current practice. Given the finding that a minority of patients with rLA and rLR disease received adjuvant RT, it is possible that the relationship observed is due to indication bias and referral bias where patients with higher risk and poorer prognostic features were preferentially referred for consideration of adjuvant RT. Key pathologic features such as histology, PNI, margin status, and extranodal extension among others, were not available for analysis. These are known prognostic features that would be important to consider when attempting to draw conclusions on the efficacy of RT for endpoints such as overall survival.

Previous cancer diagnosis within the past 5 years was also a prognostic of poor outcome. Previous cancer diagnosis and treatment is linked to increased frailty, and has been shown to be associated with poorer outcomes [33,34]. Other metachronous cancers may also be more prone to metastasizing and leading to death than NMSCs [35]. In both rLA and rLR disease, female sex and younger age was associated with a decreased HR for OS. This is in agreement with previously published population data for all NMSCs [12,13,36].

The disease state of rLA or rLR disease was not associated with a statistically significant HR for any recurrence over time with a HR of 0.95. There are multiple potential explanations for this finding. Either rLA and rLR disease behave similarly with regard to recurrence rates, the preferential patterns towards adjuvant RT for rLR may be attenuating recurrence rates, or the true proportion of locoregional recurrences is not well captured in current databases. A large proportion of recurrences may have been biopsied without resection, treated for salvage without pathologic confirmation of recurrence, or may not be identified prior to death from other cause. These cases are not captured in our databases.

### 4.3. Radiation Oncology Consultations

The incidence of resected locally advanced and locoregional disease increased during the study period; however, it was not routine for patients with either rLA or rLR disease to be seen by a radiation oncologist. Only 29.6% of patients with rLA disease were seen by radiation oncology, where 50.7% of patients with rLR disease received a radiation oncology consultation. The decreased rate of rLA consultation compared to rLR could be because the strength of recommendation for adjuvant RT in resected locally advanced basal cell carcinomas is limited [2]. However, current guidelines suggest strong recommendations for adjuvant radiation in most patients with involved lymph nodes (rLR disease) after therapeutic lymphadenectomy in both squamous cell carcinoma and basal cell carcinoma, with the exception of patients with a single, small lymph node without extracapsular extension [2]. Despite this guidance, not all rLR disease was referred to radiation oncology in our study. Due to the lack of histologic specificity and pathologic information in the ICES database used for this study, it is unclear if histology or pathology could play a role in decision making for referrals.

### 4.4. Receiving Adjuvant Radiation

There was a low proportion of patients with rLA and rLR H&N NMSC who received adjuvant RT over the study period with utilization rates of 19.4% and 37.9%, respectively. There was a decreasing trend, but no statistically significant change, in the proportion of patients receiving adjuvant radiation in either group. rLR disease had a higher relative OR (2.85) to receive adjuvant RT compared to rLA disease, which is expected given the stronger recommendation for adjuvant RT in patients with involved lymph nodes [2]. Population-level data for adjuvant RT in H&N NMSC is relatively sparse. One study of two tertiary centers in the United States found that adjuvant RT was delivered to 54.7% of the study population [25].

In both rLA or rLR disease, non-rural demographic was associated with increased odds of receiving adjuvant radiation. This suggests that various socioeconomic factors may be potential barriers to the receipt of adjuvant RT. While rural demographic was associated with a decreased likelihood of receiving adjuvant RT, the distance to the nearest regional cancer center did not significantly change the receipt of adjuvant RT. Neither rural habitation nor distance to a cancer center impacted access to the initial radiation consultation. In combination, this suggests that while patients were able to attend the consultation, there are access barriers specific to rural patients that exist other than travel distance alone. The logistics of multiple, daily scheduled fractions of radiation over many weeks may pose barriers (time off work, childcare, etc.) for this population. Hypofractionated or ultra-hypofractionated adjuvant regimens of RT for H&N NMSC may benefit these populations and could be the topic of future studies.

### 4.5. Limitations

Comprehensive data records are limited for NMSCs because of the logistical difficulties in capturing the volume of such a prevalent disease. Databases such as the Ontario Cancer Registry have similar difficulties [37,38]. The Ontario Cancer Registry derives data from in-hospital diagnoses, pathology results, death certificates, and cancer center referrals, and do not capture procedures done in clinic or private office settings [38]. Although the dataset in this study reports 11,308 cases of H&N NMSC from 2003 to 2019, current data collection methods certainly underrepresent the true incidence of NMSCs. Information about pre-invasive and early-stage skin cancer is not routinely collected in the Ontario Cancer Registry and the vast majority of cases are not managed within the hospital setting and therefore were not the focus of this study. This study focuses on surgically resected, very advanced disease because major surgical resections would be required to be completed in hospitals with public single-payer billing codes that could be tracked and collated. Similarly, any radiation oncology consultation and treatment would be available through OHIP, strengthening our ability to reliably identify our defined study population.

Because pathologic staging was not available in the ICES datasets, we utilized surgical billing codes as a surrogate for pathologic staging. This allows for the misclassification and upstaging of T or N stage. For example, smaller tumors that may have required more complicated surgeries for closure due to cosmesis or wound management could be upstaged as T3 or 4. Similarly, neck dissections that yielded no pathologically involved nodes were classified as node-positive based on our assumptions. This could falsely increase the incidence of more locally advanced disease, subsequently improve the survival estimates.

When defining our local recurrence data, disease with second surgical billing code (Appendix A) was used to identify recurrence. A proportion of local or regional recurrences, however, would have been simply biopsied alone, treated with radiation as opposed to surgery or even palliated with the best supportive care. These patient outcomes are not well captured in the available databases. Therefore, our recurrence statistics likely underrepresent the true incidence, but are the best available estimation given the limitations of the databases housing NMCSs. Similarly, many of the databases used were not specifically created for research purposes. This introduces the possibility that the data may have some inaccuracies present. Because the ICES database does not contain cause of death, we were unable to calculate metrics such as cancer-specific survival. The majority of our study population consisted of individuals aged 60 or older, who often present with competing comorbidities, such as cardiovascular disease. A limitation to this study is the inability to identify whether mortality was attributed to the index cancer or other comorbidities, thus decreasing our recurrence free-survival estimates.

In addition, the reasons for non-referral of patients are not available. It is possible that patients refused, were not medically fit for further therapy or that the surgeon felt that adjuvant therapy was not required. These rationales were not captured in the ICES databases. The retrospective nature of this study also limits the ability to describe causality of our findings.

The treatment paradigm of very advanced NMSC is changing rapidly. Although systemic therapy was not a standard of care during the study period, newer targeted therapy and immunotherapies have now demonstrated efficacy in the setting of very advanced non-melanoma skin cancers [39,40,41]. As a result, current skin cancer care pathways recommend that patients with locally advanced, node-positive or recurrent NMSC should be considered for radiation and systemic treatments [42]. The results of this study may help inform the need for both medical and radiation oncology resources for this patient population.

## 5. Conclusions

This study aimed to characterize the incidence, survival outcomes, and radiation oncology referral or treatment patterns of resected, locally advanced and lymph node-positive non-melanoma skin cancers of the head and neck in Ontario, Canada from 2003 to 2019. The study found an increasing incidence of rLA disease with no increase in rLR disease. Despite the international consensus that patients with node-positive disease should receive adjuvant RT to improve locoregional control, the proportion of patients with rLA and rLR H&N NMSCs receiving consultation remains low at 29.6% and 50.7%, respectively. Female sex and younger age patients had improved OS; however patients receiving adjuvant therapies (systemic or radiation) also had poorer OS, which is likely a function of more severe disease triggering consultation. Given advances in systemic therapies, consideration should be given to establishing multi-disciplinary skin cancer clinics with surgical, radiation and medical oncologists for patients with the highest risk factors at the outset to determine the optimal treatment paradigm for each patient. Overall, these findings reveal a growing burden of rLA and rLR disease and low referral rates for radiation oncology despite guideline recommendations. This highlights a need for further evaluation of referral practices and guideline adherence in order to optimize evidence-based cancer care.

## Figures and Tables

**Table 1:** Patient characteristics by group of resected head and neck non-melanoma skin cancer.

		Resected Early-Stage Disease	Resected Locally Advanced (rLA) Disease	Resected Locoregional (rLR) Disease
***N***		4111	2962	1055
Year of Diagnosis, *N* (%)	2003–2008	1059 (25.8)	573 (19.4)	405 (38.5)
	2009–2014	1553 (37.8)	1038 (35.0)	367 (34.9)
	2015–2019	1499 (36.5)	1351 (45.7)	282 (26.9)
Sex, *N* (%)	Male	2578 (62.7)	1942 (65.6)	716 (67.9)
Age Groups, *N* (%)	18–39	271 (6.6)	178 (6.1)	128 (12.1)
	40–59	789 (19.3)	517 (17.5)	315 (29.8)
	60–79	1814 (44.2)	1382 (46.7)	486 (46.1)
	80+	1237 (30.2)	885 (29.9)	126 (12.0)
Anatomic Location of Primary *, *N* (%)	Lip	61 (1.5)	13 (0.4)	19 (1.8)
	Eyelid, including cantus	151 (3.7)	39 (1.3)	12 (1.1)
	Ear	513 (12.5)	207 (7.0)	167 (15.8)
	Other and Unspecified parts of Face	2184 (53.1)	1534 (51.8)	433 (41.0)
	Scalp and Neck	1202 (29.2)	1169 (39.5)	424 (40.2)
Multiple Cancers at Diagnosis, *N* (%)	Yes	67 (1.6)	57 (1.9)	45 (4.3)
Prior Cancer Within 5 Years of Diagnosis, *N* (%)	Yes	238 (5.8)	199 (6.7)	53 (5.0)
Prior Head and Neck Cancer in 5 Years of Diagnosis, *N* (%)	Yes	16 (0.4)	27 (0.9)	16 (1.5)
Had Radiation Oncology Consult *N* (%)	Prior to Surgery	80 (2.0)	157 (5.3)	185 (17.5)
	Post-Surgery †	985 (24.0)	847 (28.6)	493 (46.7)
	Any Time †	999 (24.3)	876 (29.6)	535 (50.7)
Received Radiation, *N* (%)	Adjuvant †	666 (16.2)	574 (19.4)	400 (37.9)

† Within 60 days after surgery. * Based on ICD-10 codes in Appendix B.

**Table 2:** Patient outcomes by group of resected head and neck non-melanoma skin cancer.

		Resected Early-Stage Disease	Resected Locally Advanced (rLA) Disease	Resected Locoregional (rLR) Disease
*N*		4111	2962	1055
Overall Survival, *N* (%)	Deaths	1495 (36.4)	1049 (35.4)	431 (60.8)
	Median survival (years) (95% CI)	11.3 (10.6, 12.2)	10.2 (9.3, 11.5)	12.8 (11.1, 14.2)
	5-year OS% (95% CI)	74 (72, 75)	69 (67, 71)	68 (65, 71)
Recurrence-Free Survival from Day of Surgery, *N* (%)	Events		1489 (50.3)	562 (53.3)
	Median survival (years) (95% CI)		5.4 (4.9, 5.8)	6.2 (5.2, 7.6)
	5-year (95% CI)		52 (49, 54)	54 (51, 57)
Recurrence Status Stratified by Recurrence Type, *N* (%)	Local	57 (1.4)	500 (16.9)	97 (9.2)
	Regional	12 (0.3)	115 (3.9)	75 (7.1)
	Local and Regional	13 (0.3)	121 (4.1)	61 (5.8)
	Death without Recurrence	1458 (35.5)	753 (25.4)	329 (31.2)
	Alive, no events	2571 (62.5)	1473 (49.7)	493 (46.7)

**Table 3:** Univariate and multivariable prognostic factors of overall survival, from landmark date of 90 days post surgery, by group of resected head and neck non-melanoma skin cancer.

		Univariable Results		Multivariable Results	
Factor	Comparator	Hazards Ratio (95% CI)	*p*-Value	Hazards Ratio (95% CI)	*p*-Value
**Resected Locally Advanced (rLA) Disease**					
Year of Diagnosis	year	1.04 (1.02, 1.06)	<0.001	1.01 (0.99, 1.03)	0.26
Sex	F vs. M	0.69 (0.61, 0.79)	<0.001	0.77 (0.67, 0.89)	<0.001
Age Groups	group	1.43 (1.39, 1.48)	<0.001	1.46 (1.41, 1.51)	<0.001
Anatomic Location of Primary *	Lip	1.65 (0.78, 3.49)	0.017	1.70 (0.79, 3.64)	0.002
	Eyelid, including cantus	1.17 (0.75, 1.83)		0.93 (0.58, 1.50)	
	Ear	0.74 (0.56, 0.96)		0.79 (0.60, 1.03)	
	Other and Unspecified parts of Face	0.86 (0.76, 0.98)		0.78 (0.68, 0.90)	
	Scalp and Neck	Reference		Reference	
Multiple Cancers at Diagnosis	Yes vs. No	1.91 (1.30, 2.80)	<0.001	1.22 (0.82, 1.80)	0.33
Prior Cancer Within 5 Years	Yes vs. No	1.92 (1.56, 2.36)	<0.001	1.29 (1.04, 1.60)	0.019
Any Systemic Treatment	Yes vs. No	1.58 (1.35, 1.84)	<0.001	1.67 (1.40, 2.00)	<0.001
Adjuvant Radiation	Yes vs. No	2.39 (2.11, 2.72)	<0.001	1.67 (1.45, 1.92)	<0.001
**Resected Locoregional (rLR) Disease**					
Year of Diagnosis	year	1.01 (0.98, 1.03)	0.57	0.98 (0.95, 1.01)	0.23
Sex	F vs. M	0.62 (0.50, 0.77)	<0.001	0.73 (0.58, 0.91)	0.006
Age Groups	group	1.23 (1.19, 1.28)	<0.001	1.25 (1.20, 1.30)	<0.001
Anatomic Location of Primary *	Lip	0.61 (0.25, 1.48)	0.095	0.65 (0.26, 1.60)	0.39
	Eyelid, including cantus	0.71 (0.27, 1.92)		1.22 (0.45, 3.36)	
	Ear	0.67 (0.50, 0.90)		0.75 (0.55, 1.03)	
	Other and Unspecified parts of Face	0.89 (0.73, 1.10)		0.94 (0.75, 1.17)	
	Scalp and Neck	Reference		Reference	
Multiple Cancers at Diagnosis	Yes vs. No	1.14 (0.68, 1.92)	0.61	0.63 (0.37, 1.05)	0.075
Prior Cancer Within 5 Years	Yes vs. No	2.31 (1.57, 3.39)	<0.001	1.78 (1.19, 2.68)	0.006
Any Systemic Treatment	Yes vs. No	2.07 (1.70, 2.52)	<0.001	1.73 (1.38, 2.16)	<0.001
Adjuvant Radiation	Yes vs. No	3.44 (2.83, 4.19)	<0.001	2.57 (2.05, 3.21)	<0.001

* Based on ICD-10 codes in Appendix B.

**Table 4:** Univariate and multivariate model of predictive factors of radiation oncologist consultation.

		Univariate Results		Multivariate Results	
Factor	Comparator	Odds Ratio (95% CI)	*p*-Value	Odds Ratio (95% CI)	*p*-Value
**Resected Locally Advanced (rLA) Disease (*****n*** **= 2951)**					
Year of Diagnosis	year	1.01 (0.99, 1.03)	0.26	0.93 (0.91, 0.95)	<0.001
Sex	F vs. M	0.77 (0.67, 0.89)	<0.001	0.84 (0.70, 1.01)	0.068
Age Groups	group	1.46 (1.41, 1.51)	<0.001	1.07 (1.04, 1.11)	<0.001
Anatomic Location of Primary *	Lip	1.70 (0.79, 3.64)		3.46 (1.07, 11.22)	<0.001
	Eyelid, including cantus	0.93 (0.58, 1.50)		1.70 (0.87, 3.34)	
	Ear	0.79 (0.60, 1.03)	0.002	0.76 (0.54, 1.08)	
	Other and Unspecified parts of Face	0.78 (0.68, 0.90)		0.71 (0.59, 0.85)	
	Scalp and Neck	Reference		Reference	
Multiple Cancers at Diagnosis	Yes vs. No	1.22 (0.82, 1.80)	0.33	4.61 (2.58, 8.26)	<0.001
Prior Cancer Within 5 Years of Diagnosis	Yes vs. No	1.29 (1.04, 1.60)	0.019	1.08 (0.78, 1.50)	0.64
**Resected Locoregional (rLR) Disease (*****n*** **= 1045)**					
Year of Diagnosis	year	0.98 (0.95, 1.01)	0.23	0.99 (0.96, 1.02)	0.5
Sex	F vs. M	0.73 (0.58, 0.91)	0.006	0.81 (0.61, 1.07)	0.13
Age Groups	group	1.25 (1.20, 1.30)	<0.001	1.09 (1.04, 1.13)	<0.001
Anatomic Location of Primary *	Lip	0.65 (0.26, 1.60)	0.39	1.14 (0.42, 3.06)	0.28
	Eyelid, including cantus	1.22 (0.45, 3.36)		1.59 (0.45, 5.62)	
	Ear	0.75 (0.55, 1.03)		0.70 (0.47, 1.03)	
	Other and Unspecified parts of Face	0.94 (0.75, 1.17)		0.81 (0.61, 1.10)	
	Scalp and Neck	Reference		Reference	
Multiple Cancers at Diagnosis	Yes vs. No	0.63 (0.37, 1.05)	0.075	3.45 (1.57, 7.57)	0.002
Prior Cancer Within 5 Years of Diagnosis	Yes vs. No	1.78 (1.19, 2.68)	0.006	1.31 (0.69, 2.49)	0.41

* Based on ICD-10 codes in Appendix B.

**Table 5:** Multivariate model of prognostic factors of receipt of adjuvant radiation treatment.

Factor	Comparator	Odds Ratio (95% CI)	*p*-Value
Year of Diagnosis	year	1.01 (0.99, 1.03)	0.5
Sex	F vs. M	1.00 (0.80, 1.24)	0.97
Age Groups	group	1.08 (1.05, 1.12)	<0.001
Rural	No vs. Yes	1.52 (1.09, 2.12)	0.013
Multiple Cancers at Diagnosis	Yes vs. No	2.20 (1.36, 3.56)	0.001
Prior Cancer Within 5 Years of Diagnosis	Yes vs. No	1.41 (0.97, 2.04)	0.073
Surgical Group	LR disease vs. LA disease	2.85 (2.27, 3.57)	<0.001

## Data Availability

Data was collected from the ICES database and can be found here (https://www.ices.on.ca/about-ices-data/ (accessed on 15 June 2022)). Please refer to our methods section for a discussion of specific databases used.

## Abbreviations

The following abbreviations are used in this manuscript:

H&N NMSC	Head and neck non-melanoma skin cancer
NMSC	Non-melanoma skin cancer
rLA	Resected locally advanced
rLR	Resected locoregional
BCC	Basal cell carcinoma
SCC	Squamous cell carcinoma
ASTRO	American Society for Radiation Oncology
ICD-10	International Classification of Diseases 10
ICES	Institute for Clinical and Evaluative Sciences
OHIP	Ontario Health Insurance Plan
TNM	Refers to Tumor, Nodes, Metastasis staging system
OS	Overall Survival
RFS	Recurrence-Free Survival
RT	Radiotherapy
OR	Odds Ratio
HR	Hazard Ratio

## Appendix A

### Appendix A.1. Locoregional Disease Surgery Codes (N+) Codes

OHIP Neck dissection codes:
R910—limited dissection, must include 2 levels (unilateral) or central compartment
R915—comprehensive dissection, must include 3 or more levels, unilateral
OHIP Parotidectomy codes:
S043—Total (with preservation of facial nerve)
S044—Total (without preservation of facial nerve)
S045—Subtotal (with preservation of facial nerve)
S047—Repeat subtotal (with preservation of facial nerve)

### Appendix A.2. Locally Advanced Surgery Code (T3-4 N0s) Codes

OHIP Skin flap codes:
Rotations, transpositions, Z-plasties of face, neck or scalp
R047—Defect 5.1 to 10 cm average diameter
R076—Defect more than 10 cm average diameter
OHIP Advancement flap codes:
R012—Defect 5.1 to 10 cm
OHIP Split thickness grafts codes:
R087—Major, complex areas, e.g., face, neck, hands
R088—Extensive major, very large areas
OHIP Free flap islands codes:
R064—Elevation of free island skin and subcutaneous flap and closure of defect
R067—Elevation of innervated free island skin and subcutaneous flap and closure of defect
OHIP Free island flaps codes:
R134—Elevation of free island skin and bone flap and closure of defect
R125—Elevation of free island skin and muscle flap and closure of defect
OHIP Myocutaneous, myogenous or fascia-cutaneous flaps codes:
R005—Sterno-mastoid, tensor fascia lata, gluteus maximus, gracilis, sartorius, rectus femoris, gastrocnemius (medial and lateral), trapezius
R006—Pectoralis major
R155—Latissimus dorsi or unilateral rectus abdominus
R091—Full thickness graft to the eyelid, nose, lip, face
R081—Moh’s micrographic surgery

### Appendix A.3. Early-Stage (T1-2N0) Codes

R010—malignant melanoma wide excision
R011—advancement flaps 2.1 to 5 cm of the face, neck or scalp
R045—Rotations, transpositions, Z-plasties of face, neck of scalp less than 2 cm average diameter
R046—Defect 2.1 to 5 cm average diameter
Malignant lesion codes:
R048—simple excision of face or neck—single
R049—simple excision of face or neck—two lesions
R050—simple excision of face or neck—3 or more lesions
Premalignant codes:
R160—Face or neck simple excision—single lesion
R161—Face or neck simple excision—2 lesions
R162—Face or neck simple excision—3 or more lesions
Curettage, electrodesiccation or cryosurgery—face or neck codes:
R018—Single lesion
R019—Two lesions
R020—Three or more lesions
R051—Laser surgery on premalignant and malignant lesions
Z117—chemical and/or cryotherapy treatment of skin lesions
Premalignant—simple excision codes:
R163—single lesion
R164—two lesions
R165—three or more lesions
Malignant—simple excision codes:
R094—single lesion
R040—two lesions
R041—three or more lesions
Curettage, electrodesiccation or cryosurgery codes:
R031—single lesion
R032—two lesions
R033—three or more lesions

## Appendix B

### Histology Codes of Index Cancer Definitions

C44.0—Skin of lip
C44.1—Skin of eyelid, including canthus
C44.2—Skin of ear and external auricular canal
C44.3—Skin of other and unspecified parts of face
C44.4—Skin of scalp and neck
